# Compound 275# Induces Mitochondria-Mediated Apoptosis and Autophagy Initiation in Colorectal Cancer Cells through an Accumulation of Intracellular ROS

**DOI:** 10.3390/molecules28073211

**Published:** 2023-04-04

**Authors:** Dong-Lin Yang, Yong Li, Shui-Qing Ma, Ya-Jun Zhang, Jiu-Hong Huang, Liu-Jun He

**Affiliations:** 1 College of Pharmacy, National & Local Joint Engineering Research Center of Targeted and Innovative Therapeutics, Chongqing Key Laboratory of Kinase Modulators as Innovative Medicine, Chongqing University of Arts and Sciences, Chongqing 402160, China; 2 College of Pharmaceutical Sciences and Chinese Medicine, Southwest University, Chongqing 400715, China

**Keywords:** compound 275#, CRC, apoptosis, ROS, autophagy

## Abstract

Colorectal cancer (CRC) is the most common intestinal malignancy, and nearly 70% of patients with this cancer develop metastatic disease. In the present study, we synthesized a novel compound, termed N-(3-(5,7-dimethylbenzo [d]oxazol-2-yl)phenyl)-5-nitrofuran-2-carboxamide (compound 275#), and found that it exhibits antiproliferative capability in suppressing the proliferation and growth of CRC cell lines. Furthermore, compound 275# triggered caspase 3-mediated intrinsic apoptosis of mitochondria and autophagy initiation. An investigation of the molecular mechanisms demonstrated that compound 275# induced intrinsic apoptosis, and autophagy initiation was largely mediated by increasing the levels of the intracellular accumulation of reactive oxygen species (ROS) in CRC cells. Taken together, these data suggest that ROS accumulation after treatment with compound 275# leads to mitochondria-mediated apoptosis and autophagy activation, highlighting the potential of compound 275# as a novel therapeutic agent for the treatment of CRC.

## 1. Introduction

Colorectal cancer (CRC), an aggressive primary gastrointestinal malignancy, has the third-highest incidence and second-highest mortality in all types of cancers worldwide. The number of CRC cases increases annually, and CRC poses a serious threat to human life and health [1]. Due to unknown etiology, lack of obvious symptoms in the early stage, and high level of metastasis, most CRC patients are diagnosed at advanced stages and have high mortality [2]. Despite the recent progress that has been made in treatment, the clinical outcomes and prognoses of patients with advanced-stage CRC remain extremely poor [3]. Thus, novel treatment regimens, such as the development of novel drugs that target proliferating tumor cells, may help to prolong the survival of CRC patients.

Reactive oxygen species (ROS) are a group of short-lived and highly reactive byproducts generated by aerobic metabolisms, which consist of hydroxyl radicals (OH^•^), superoxide (^•^O_2_^−^), hydroperoxy1 (HO_2_^•^) radicals, and other nonradical members, such as hydrogen peroxide (H_2_O_2_) and singlet oxygen [4,5]. Cellular ROS are mainly originated from subcellular compartments or organelles, such as mitochondria, endoplasmic reticulum, lysosomes, and peroxisomes, by enzymatic reactions involving cyclooxygenases, oxidoreductases, NADPH oxidases, xanthine oxidases, and lipoxygenases and through the iron-catalyzed Fenton reaction [6]. As excellent significant intracellular signaling molecules, ROS play an important role in several physiological and pathological processes [7]. It is generally believed that low and moderate levels of ROS are required to promote tumor proliferation, survival, and metastasis [8,9]. In contrast, excessive ROS can lead to cellular oxidative stress and cell malfunction, which cause damage to DNA, proteins, or lipids, finally resulting in apoptotic or necrotic cell death [7,10]. Therefore, the identification of novel anticancer agents, which induce apoptotic cell death through the ROS generation mechanism, may be a necessary and effective treatment strategy for CRC.

In fact, many cancer therapeutic drugs often eliminate cancer cells and drug resistance through elevating ROS generation [11,12,13]. The aim of the present study was to synthesize a novel, small-molecule compound for potential future medical use, which is capable to induce excessive intracellular ROS. We investigated the anticancer activity of the novel, synthesized compound 275# against CRC in vitro and illustrated the underlying mechanism. Our data demonstrate that compound 275# suppresses the proliferation of CRC and acts as an inducer of ROS generation. Interestingly, ROS accumulation induced by compound 275# further triggered mitochondria-mediated intrinsic apoptosis and initiated autophagy, implying that compound 275# is a promising candidate as an anticancer agent.

## 2. Results

### 2.1. Synthesis of Compound 275#

**Figure 1:**
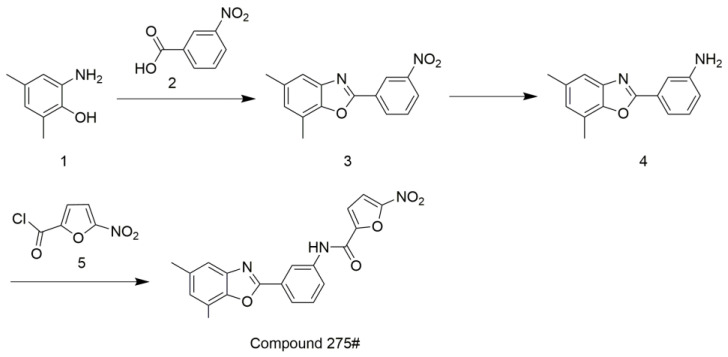
Strategy for the synthesis of compound 275#.

The synthesis of compound 275# is indicated in Figure 1. Compound 1 (1 equivalents (equiv)) and compound 2 (1 equiv) were placed in PPA at 60 °C. The mixture was then heated at 100 °C for 1 h and followed at 125 °C for an additional 1.5 h. After cooling the mixture to room temperature, water was added, and the mixture was carefully neutralized with solid NaOH to pH~7.5–8.5. The formed precipitate was filtered off, suspended in 15% aqueous NaOH, filtered, washed with water, and dried. Crystallization from ethanol gave compound 3 in a 52% yield.

Compound 3 (1 equiv) was dissolved in hot CH_3_OH. Then, ammonium formate (10 equiv) was added into the mixture followed by the portion-wise addition of a suspension of Pd/C (10%) (0.1 equiv) in water. The mixture was refluxed for 1 h, cooled to room temperature, filtered through celite, and concentrated in vacuo. The residue was suspended in water and extracted with ether. The combined ethereal extracts were dried over anhydrous Na_2_SO_4_ and concentrated to provide compound 4 in a yield of 90%.

The mixture of compound 4 (1 equiv) and acid chloride 5 (1.1 equiv) in dry DMF triethylamine (1.2 equiv) was added and stirred at 70 °C for 8 h. After quenching the reaction mixture with 3% Na_2_CO_3_, the precipitated product was filtered, washed with water, and dried. The product was recrystallized from the EtOH–EtOAc mixture to provide the target compound 275# in a 60% yield (Figure S1, Supplementary Materials).

### 2.2. Compound 275# Exhibits Cytotoxic Effects against CRC Cells

**Figure 2:**
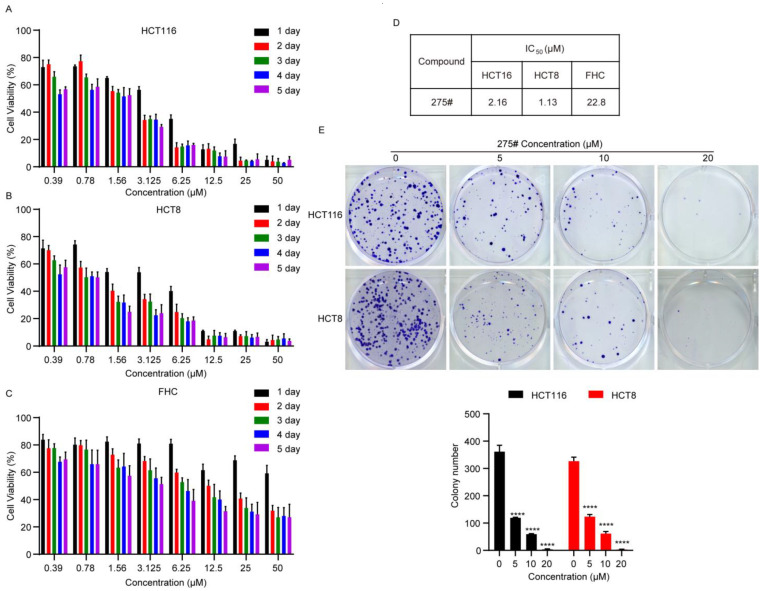
Compound 275# suppresses the proliferation and viability of CRC cells. (**A**,**B**) HCT116 and HCT8 cells were treated with the indicated concentrations of compound 275# for 1, 2, 3, 4, and 5 days. Relative cell viability was measured with MTT assay. (**C**) The cytotoxicity and specific effect of compound 275# on normal human rectal mucosal cells (FHC). (**D**) Calculated IC_50_ values of compound 275# for HCT116, HCT8, and FHC cell lines. (**E**) Colony formation assay was performed to evaluate growth in vitro after treatment with DMSO, 5, 10, and 20 μM compound 275# for 14 days. The colonies were visualized with the images. All data are presented as the mean ± SD of three independent experiments. **** *p* < 0.001 versus vehicle.

To explore the cytotoxicity of the novel, small-molecular compound 275# in CRC cells, an MTT assay was performed after exposure to this compound. As shown in Figure 2A,B, compound 275# dramatically reduced the cell viability in HCT116 and HCT8 cell lines in a dose- and time-dependent manner. In comparison, treatment with compound 275# was associated with a lower cytotoxicity in normal adult colonic epithelial cell line FHC (Figure 2C). Corresponding half-maximal inhibitory concentration (IC_50_) values of compound 275 for the cell lines are shown in Figure 2D. To further examine its inhibitory effects, CRC cells were treated with different concentrations of compound 275# for 14 days, and the relationship between cell growth and concentration was evaluated. The colony formation assay showed that cells exposed to compound 275# exhibited markedly decreased cell growth in a dose-dependent manner, as convinced by the smaller colonies and reduced colony numbers compared with the control group (Figure 2E). Taken together, these results show that compound 275# could suppress cell proliferation and growth, suggesting that it has the potential as an inhibitor for the treatment of human CRC.

### 2.3. Compound 275# Promotes ROS Accumulation and ER Stress in CRC Cells

**Figure 3:**
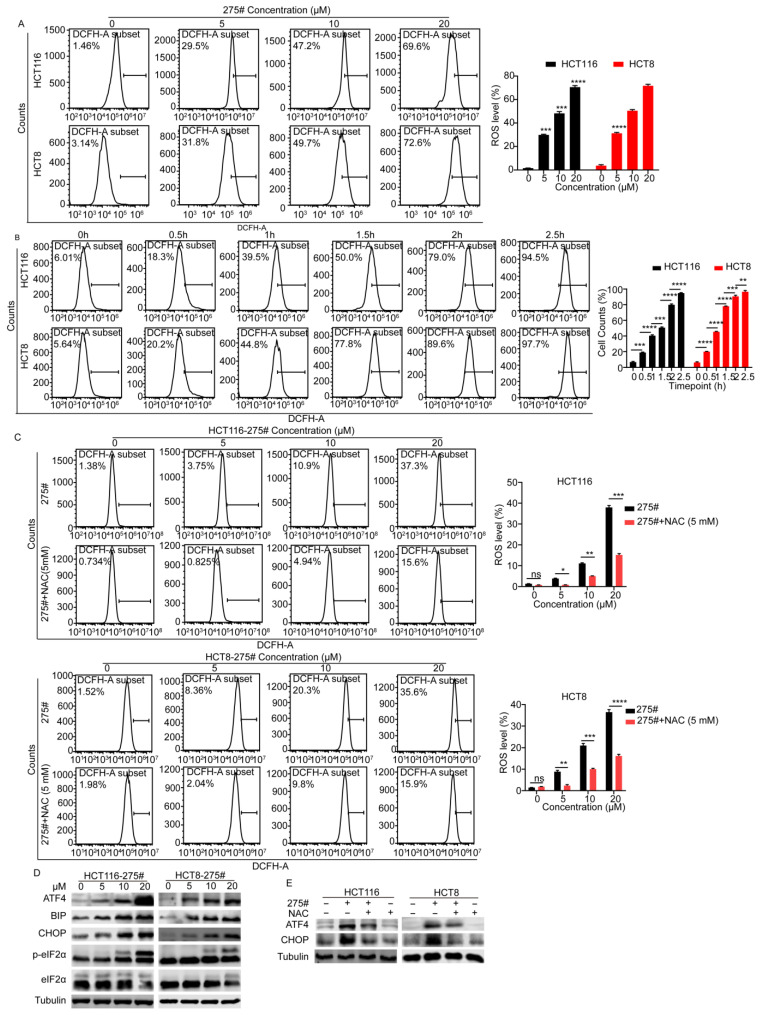
Compound 275# induces the accumulation of ROS and promotes ROS-dependent ER stress. (**A**) Compound 275# triggers ROS generation in a dose-dependent manner. HCT116 and HCT8 cells were treated with the indicated concentrations of compound 275# for 2 h and then were exposed to DCFH-DA for another 20 min. Flow cytometry analysis was conducted to assess ROS generation. (**B**) Compound 275# triggers ROS generation in a time-dependent manner. (**C**) NAC, a potent antioxidant and ROS scavenger, was capable to reduce compound 275#-induced ROS accumulation in CRC cells. HCT116 and HCT8 cells were pretreated with or without NAC (5 mM) for 1 h and subsequently treated with compound 275# for 24 h before staining with DCFH-DA (10 µM) for 20 min. (**D**) Following treatment with different concentrations of compound 275#, cells were lysed, and expression of ER stress-related proteins was examined by Western blotting. (**E**) NAC can reverse compound 275#-triggered ER stress. β-tubulin was used as the loading control. Data are presented as the mean ± standard deviation of three independent experiments. * *p* < 0.05, ** *p* < 0.01, *** *p* < 0.001, and **** *p* < 0.0001 vs. control.

To gain insight into the action mode exhibited by compound 275# in CRC proliferative inhibition, we examined the intracellular ROS generation by flow cytometry after incubation with the specific ROS-detecting fluorescent dye (DCFH-DA) in HCT116 and HCT8 cells. As indicated in Figure 3A, ROS levels were considerably elevated in cells exposed to different concentrations of compound 275# when compared with control groups. In response to compound 275#, we found a time-dependent increase in intracellular ROS levels, and the earliest generation was detected at 0.5 h post treatment in CRC cells (Figure 3B). Next, flow cytometry indicated that pretreating the cells with NAC, a potent antioxidant and ROS scavenger, was capable to significantly reduce the number of DCFH-DA-positive HCT116 and HCT8 cells (Figure 3C). To further detect whether ROS specifically originate from mitochondria, MITOSOX, a novel fluorescent probe that specifically targets mitochondria in living cells, was used and found that most of ROS induced by compound 275# mainly originate from mitochondria (Figure S2, Supplementary Materials).

In light of previous studies showing that ROS accumulation is closely associated with the development of endoplasmic reticulum (ER) stress [14,15], we detected ER stress-related proteins in both HCT116 and HCT8 cells. As expected, compound 275# significantly increased the phosphorylation of eIF2α at Ser51 and the expression levels of several unfolded protein response (UPR)-associated proteins, such as BIP, CHOP, and ATF4, in a dose-dependent manner (Figure 3D), whereas NAC reduced its ability to upregulate ATF4 and CHOP (Figure 3E), suggesting that ER stress induced by compound 275# is dependent on excessive intracellular ROS.

### 2.4. Compound 275# Induces Mitochondria-Mediated Intrinsic Apoptosis in CRC Cells

**Figure 4:**
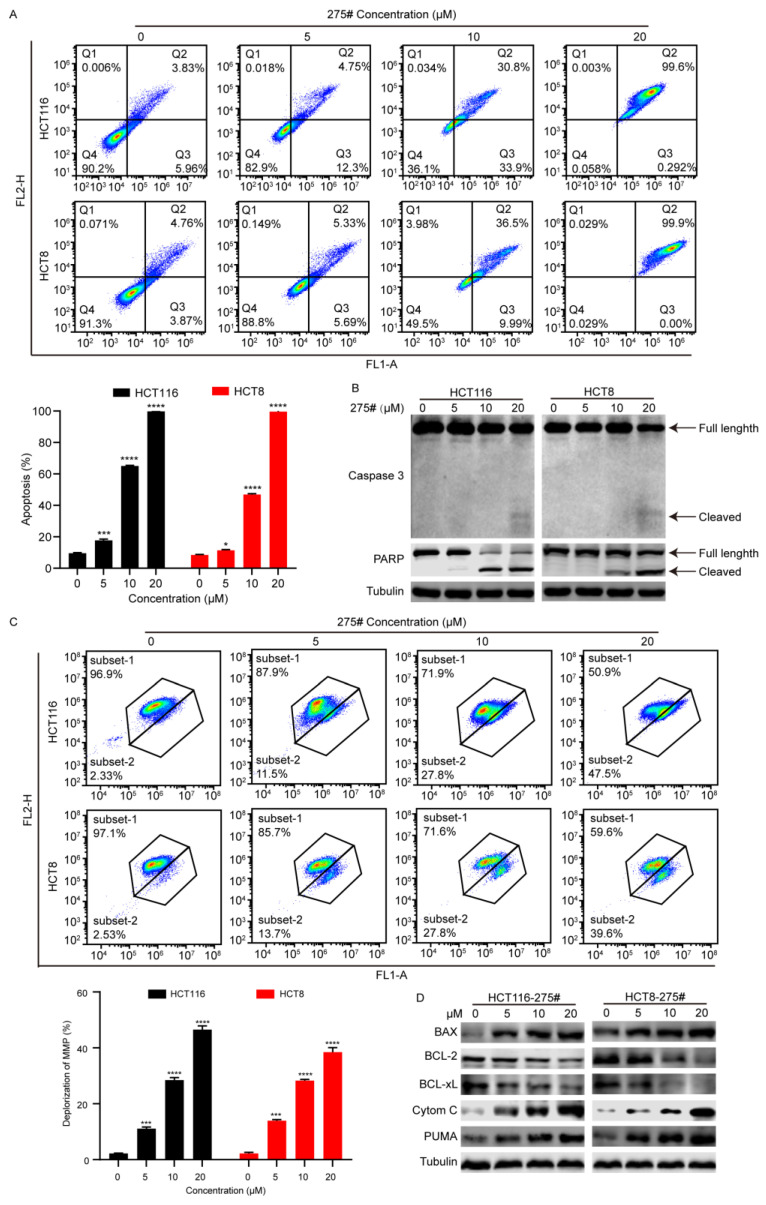
Compound 275# promotes mitochondria-mediated intrinsic apoptosis in both HCT116 and HCT8 cells. (**A**) Compound 275# triggers apoptosis in a dose-dependent manner in CRC cells. HCT116 and HCT8 cells were treated with the indicated concentrations of compound 275# for 24 h. Subsequently, cells were harvested and stained with annexin-V/PI. The color indicates the cell density and it changes from blue to red as the density increases. (**B**) Compound 275# can facilitate cleavage of caspase 3 and PARP. Cleaved forms of caspase 3 and PARP were detected using Western blotting after treatment with indicated doses of compound 275#. (**C**) Mitochondrial membrane potential (ΔΨm) is reduced after exposure to compound 275#. The ΔΨm was measured by flow cytometry after staining with fluorescent probe JC-1. Results indicating the intensity of green fluorescence show as folds of control from three independent experiments. The color indicates the cell density and it changes from blue to red as the density increases. (**D**) Expression of mitochondria-mediated apoptosis-related proteins was examined using Western blotting after treatment with indicated concentrations of compound 275#. β-tubulin was used as the loading control. * *p* < 0.05, *** *p* < 0.001, and **** *p* < 0.0001 vs. control.

A disproportional increase in ROS can induce the intrinsic apoptotic pathways mediated by mitochondria signaling [16,17,18]. Moreover, during ER stress, the level of CHOP expression is elevated, and CHOP functions to trigger mitochondria-mediated apoptosis by downregulating the prosurvival protein Bcl-2 [19]. Given the effects of compound 275# on the induction of ROS, apoptosis activation in response to compound 275# was evaluated. Of note, an Annexin V-FITC/PI assay was conducted using flow cytometry after HCT116 and HCT8 cells were exposed to compound 275# for 24 h. As indicated in Figure 4A, at a low dose of 5 μM, compound 275# induced 3.83% and 4.76% late-phase apoptosis (Annexin-V/PI double-positive cells) in HCT116 and HCT8 cell lines, respectively. At a dose of 20 μM, the percentage of both Annexin-V/PI-positive cells rose to 99.6% and 99.9%, respectively. Consistently, procaspase 3 and PARP were cleaved and activated in both HCT116 and HCT8 cells after treatment (Figure 4B), implying that compound 275# is capable to promote apoptosis.

Next, to evaluate whether the treatment with compound 275# affects depolarization of the mitochondrial membrane, the mitochondrial membrane potential (MMP, ΔΨm) was measured with a fluorescent probe JC-1 in CRC cells treated with or without compound 275#. As hypothesized, 20 μM of compound 275# caused more than a 40% decrease in ΔΨm in CRC cells in comparison with the control group (Figure 4C), suggesting that the apoptosis triggered by compound 275# might be related to the mitochondrial pathway. Subsequently, the expression of mitochondrial signaling-associated proteins was detected to determine whether mitochondria was involved in apoptosis in response to compound 275#. As indicated in Figure 4D, we found that antiapoptotic proteins, such as Bcl-2 and Bcl-XL, were significantly reduced in a dose-dependent manner after exposure to compound 275#, while the proapoptotic protein Puma (p53 upregulated modulator of apoptosis) was dramatically elevated. Furthermore, compound 275# remarkably increased cytochrome c levels in a dose-dependent manner in CRC cells (Figure 4D), indicating that compound 275# may induce the release of cytochrome c from mitochondria to cytosol. In addition, the protein level of p53 was not influenced by compound 275#, and we inferred that the proapoptotic effect of compound 275# may be not mediated by p53 (Figure S3, Supplementary Materials). These results show that compound 275# might inhibit cell proliferation by inducing a mitochondrial-dependent apoptotic pathway in CRC cells.

### 2.5. Induction of Intrinsic Apoptosis by Compound 275# Is Mediated through Excessive ROS

**Figure 5:**
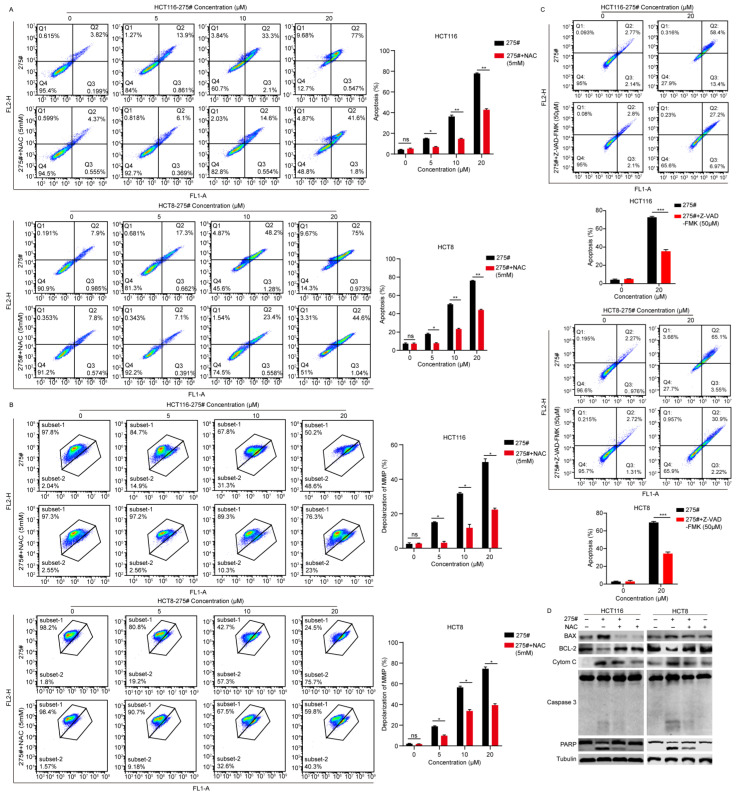
Induction of caspase-dependent intrinsic apoptosis by compound 275# is mediated through the accumulation of excessive ROS. (**A**) Blocking of ROS generation significantly decreased compound 275#-mediated apoptosis. HCT116 and HCT8 cells were pretreated with or without 5 mM NAC for 1 h and then treated with different concentrations of compound 275# for 24 h. Cell apoptosis was subsequently measured using flow cytometry. The color indicates the cell density and it changes from blue to red as the density increases. (**B**) NAC significantly reversed ΔΨm decrease induced by compound 275#. The color indicates the cell density and it changes from blue to red as the density increases. (**C**) A specific caspase 3 inhibitor, Z-VAD-FMK, significantly decreased the rate of apoptosis triggered by compound 275# in HCT116 and HCT8 cells. Both HCT116 and HCT8 cells were treated with 20 μM compound 275#, with or without the inclusion of 50 μM caspase 3 inhibitor Z-VAD-FMK before staining with PI and Annexin V. The color indicates the cell density and it changes from blue to red as the density increases. (**D**) NAC could abrogate the effects of compound 275# on the expression levels of BCL-2 family proteins, cytochrome c, and apoptotic executor caspase 3. β-tubulin was used as the loading control. Data are presented as the mean ± standard deviation of three independent experiments. * *p* < 0.05, ** *p* < 0.01, and *** *p* < 0.001 vs. control.

To further demonstrate whether the capability to induce mitochondria-mediated apoptosis by compound 275# in CRC cells was actually initiated by accumulating excessive cellular ROS, the proapoptotic effect of compound 275# on HCT116 and HCT8 cells after treatment with NAC was determined. As expected, flow cytometry showed that pretreating the cells with NAC significantly reduced the number of apoptotic cells induced by compound 275# (Figure 5A). Additionally, the effect of NAC on ΔΨm, which is reduced in response to compound 275#, was evaluated. We found that NAC pretreatment can obviously reverse the compound 275#-mediated decrease in ΔΨm in both HCT116 and HCT8 cells (Figure 5B). Subsequently, a caspase 3 inhibitor, Z-VAD-FMK, was used to detect the restorative effect on apoptosis caused by compound 275#. As shown in Figure 5C and Figure S4, Z-VAD-FMK can significantly rescue cell apoptosis induced by compound 275# and reduce the inhibitory effect of compound 275# on CRC cells. Consistently, immunoblotting results indicate that pretreating the cells with NAC dramatically downregulated the expression levels of Bax and cytochrome c and upregulated the antiapoptotic protein Bcl-2. Moreover, treatment with compound 275# alone considerably enhanced the cleavage of procaspase-3 and the downstream PARP, while combined treatment with compound 275# and NAC caused minimal proteolytic processing of procaspase-3 and cleavage of PARP (Figure 5D). Collectively, these results indicate that compound 275# induced mitochondria-mediated apoptosis in CRC cells by leading to the production of excessive ROS, which acts as a trigger of downstream caspase 3-dependent apoptosis.

### 2.6. Autophagy Initiation Triggered by Compound 275# Is Tightly Linked with Its Ability to Promote ROS Accumulation

**Figure 6:**
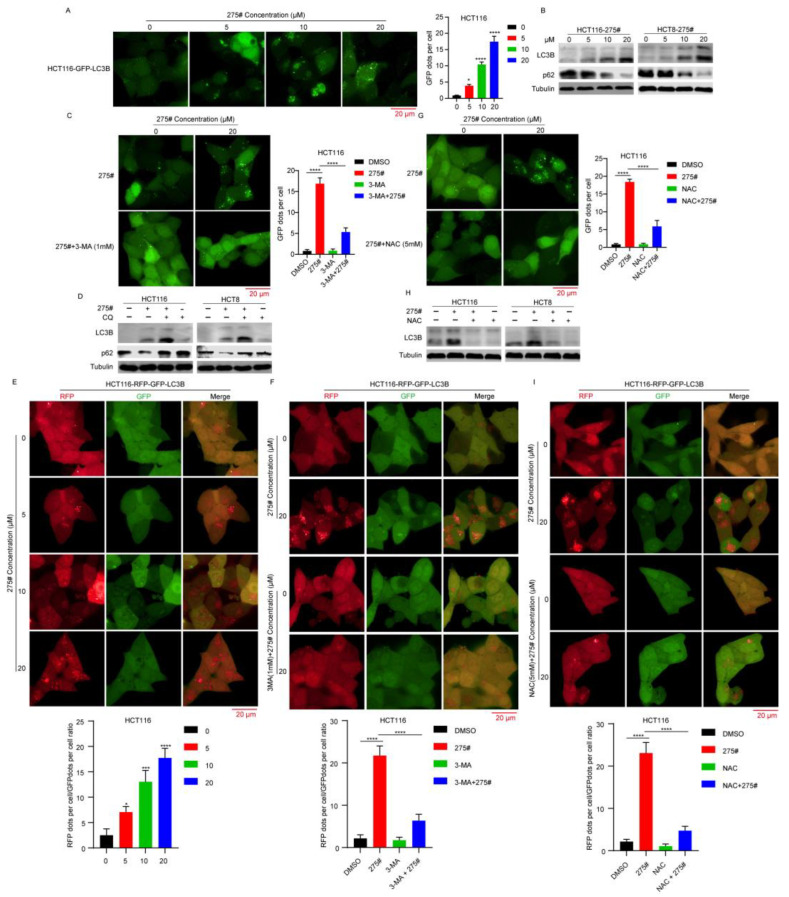
Compound 275# could promote the initiation of selective autophagy by inducing ROS accumulation. (**A**) Compound 275# markedly increased GFP-LC3B puncta. Fluorescence analysis was performed to evaluate the exogenous LC3 dot signals in HCT16 cells transfected with GFP-LC3 after treatment with or without compound 275#. Scale bar: 10 μm. (**B**) Western blotting analysis of LC3B and p62 in HCT116 cells treated with indicated concentrations of compound 275# for 12 h. β-tubulin level was used as internal load control. (**C**) 3-MA could strongly attenuate the GFP-LC3B puncta accumulation in HCT116 cells induced by compound 275#. (**D**) Compound 275# promoted LC3-II accumulation through autophagy initiation but not the inhibition of autophagic flux progression in CRC cells. HCT116 and HCT8 cells were treated with compound 202#, CQ, or a combination of them before detecting with Western blotting. (**E**) Compound 275# promoted the autophagic flux in HCT116 cells overexpressing RFP-EGFP-LC3. Scale bar, 10 μm. Yellow fluorescent dots represent the autophagosomes. Red fluorescent dots indicating autophagolysosomes were counted in three independent experiments. (**F**) Autophagy initiation effect induced by compound 275# was dramatically attenuated by cotreatment with 3-MA. (**G**) Compound 275#-induced accumulation of GFP-LC3B puncta was markedly abrogated by cotreatment with NAC. (**H**) Treatment with NAC was able to almost completely prevent the compound 275#-induced LC3-II accumulation. (**I**) NAC could greatly attenuate the enhanced autophagic flux progression caused by compound 275# treatment. Data are presented as the mean ± standard deviation of three independent experiments. * *p* < 0.05, *** *p* < 0.001, and **** *p* < 0.0001 vs. control.

Because the accumulation of ROS has been reported to exhibit a positive effect on autophagy activation [20,21], we investigated whether compound 275# can play a role in autophagy by generating excessive intracellular ROS. As indicated in Figure 6A, after exposure to compound 275#, the number of green puncta, which represent the formation of phagophores and autophagosomes, was remarkably increased in HCT116 cells transfected with the green fluorescent protein (GFP)-fused LC3B (GFP-LC3B) compared with the control group. Consistently, the autophagosome-associated form, LC3B-II, was obviously increased in CRC cells upon compound 275# treatment, whereas the level of p62, a specific marker of autophagic flux, was strongly downregulated in a dose-dependent manner (Figure 6B), indicating that autophagy was activated and autophagosomes were formed after treatment. To confirm whether or not compound 275# could regulate autophagic flux changes in CRC cells, we blocked autophagic flux by using chloroquine (CQ), a classical autophagic flux inhibitor. We found that the combined administration of both compound 275# and CQ exerted a cooperative enhancement of the LC3B-II expression level compared with either drug treatment alone. However, single-compound 275# treatment-induced p62 downregulation could be reversed by CQ (Figure 6C). Because autophagosomes can be formed by the activation of autophagy as well as by blocking autophagic flux, we next determined whether compound 275# activates or inhibits autophagy. Alternatively, 3-methyladenine (3-MA), an inhibitor of class III PI3K that inhibits autophagy initiation, was used to demonstrate compound 275#-induced autophagy. As shown in Figure 6D, 3-MA could mostly attenuate the GFP-LC3B-II puncta accumulation in CRC cells induced by compound 275#. To further confirm that compound 275# mainly plays a role in autophagy induction but not in the fusion process between autophagosome and lysosome, a tandem RFP-GFP-tagged LC3B autophagy reporter system was utilized as a dual-fluorescence pH sensor. Autophagosomes appear as yellow (both RFP and GFP signals) puncta, whereas autolysosomes appear as red (RFP-only signals) puncta. Of note, when the cells were treated with compound 275#, the number of red puncta was increased markedly without any significant increase in the number of yellow puncta (Figure 6E), whereas the autophagy promotion effect induced by compound 275# was dramatically attenuated by the cotreatment with 3-MA (Figure 6F). These results imply that compound 275# promotes fusion between autophagosome and autolysosome.

Next, cells were treated with 20 μM of compound 275# combined with 5 mM of NAC to determine the effect of NAC on autophagy. Notably, compound 275#-induced GFP-LC3B-II puncta accumulation was strongly abrogated by the cotreatment with NAC (Figure 6G). Consistently, we found that treatment with NAC was able to almost completely prevent the compound 275#-induced LC3-II accumulation (Figure 6H), implying that ROS accumulation mediated autophagy initiation in compound 275#-treated HCT116 cells. Furthermore, NAC could also greatly attenuate the enhanced autophagic flux progression caused by compound 275# treatment (Figure 6I). Collectively, these results demonstrate that excessive ROS induced by compound 275# participate in autophagy initiation.

## 3. Discussion

Chemotherapy resistance remains a major problem in limiting the efficacy of conventional and molecular-targeted cancer therapies. This problem is evident in colorectal cancer, in which, despite the advances in therapeutic options, such as EGFR inhibitors and drug combinations using EGFR inhibitor plus VEGF inhibitor/MEK inhibitor/BRAF inhibitor, the mortality rate remains high for cancer-related deaths [22,23]. Hence, there is an urgent need for therapeutic strategies to overcome drug resistance in CRC, such as the development of novel anticancer agents. In the present study, compound 275# was synthesized and exhibited some antiproliferative activity against CRC cells, facilitating further exploration of its antiproliferative effects and the underlying molecular mechanisms. Compound 275# had a structure that was similar to those previously reported benzoxazole derivatives. These derivatives possessed different inhibitory activities against cancer cells, *Trypan osoma brucei* [24] and *Visceral Leishmaniasis* [25], and selectively targeted tyrosine kinase [26], proteasome [25], and transthyretin amyloidogenesis [27]. The results from our analysis show that compound 275# can significantly inhibit CRC cell proliferation and growth, which is consistent with the above-mentioned data. However, in our current study, we failed to find the target of compound 275#. This compound may be a potential inhibitor of tyrosine kinase and proteasome, and we will demonstrate it in our future research.

It is well known that apoptosis is a fundamental regulatory process in multicellular organisms that plays a crucial role in the maintenance and development of homeostasis [28]. Moreover, apoptosis is involved in tumor formation and cancer therapy and is regarded as a main defense mechanism against tumorigenesis [29]. Numerous anticancer agents exert their inhibitory effects by triggering apoptosis [30,31,32]. It has been shown that two major apoptotic pathways are capable of initiating apoptosis: the mitochondrial-mediated intrinsic pathway and the death receptor-mediated extrinsic pathway [33]. Mitochondria-mediated apoptosis is fundamentally orchestrated by proapoptotic proteins and antiapoptotic proteins [34,35]. The production of cytochrome c from the mitochondrial intermembrane space into the cytoplasm induced by the intrinsic pathway can eventually activate the critical caspase cascade and the generation of the apoptosome, leading to the progression of apoptosis [36,37]. Accumulating evidence has indicated that certain pharmaceutical compounds cause apoptosis through the production of excessive intracellular ROS [38,39,40,41]. In agreement with these previous studies, the results of the present study show that compound 275# significantly downregulated antiapoptotic proteins, Bcl-2 and Bcl-XL, while it upregulated the proapoptotic proteins, Puma and Bax, and promoted the release of cytochrome c from mitochondria to cytosol, resulting in the decrease in ΔΨm. Moreover, these effects exerted by compound 275# can be remarkably rescued in response to NAC treatment. Based on these data, it was hypothesized that compound 275# exerted its anticancer effects on CRC cells by inducing intrinsic apoptosis following treatment, and this was due to the intracellular accumulation of ROS.

Autophagy is a self-degradative process that is associated with cell proliferation, survival, tumorigenesis, development, and stress responses [42]. Interestingly, intracellular ROS accumulation has previously been reported to initiate autophagy [43]. In this study, we found that enhanced LC-3 II was induced by compound 275# treatment in vitro, while the autophagic flux marker, p62, was markedly reduced. Moreover, the fusion between autophagosome and lysosome was facilitated after treatment with compound 275#, supported by a tandem RFP-GFP-tagged LC3B autophagy reporter system. Surprisingly, the autophagy inhibitor 3-MA blocked the compound 275#-induced accumulation of LC-3-II. However, the antioxidants NAC markedly abolished compound 275#-induced ROS generation and autophagy. The present data clearly support our hypothesis that compound 275# primarily initiates autophagy rather than the late stage of autophagy, such as the fusion between autophagosome and lysosome, and autophagy activation is triggered by ROS accumulation. Considerable evidence has indicated that apoptosis and autophagy were thought to be two mutually cross-regulated cellular events because they share several critical molecular regulators, such as JNK1, Bcl-2, and Beclin 1 [44,45,46,47]. Hence, future experiments will be undertaken to clarify the complex mutual regulatory mechanism between autophagy and apoptosis induced by compound 275#.

## 4. Materials and Methods

### 4.1. Reagents and Antibodies

Methylthiazolyldiphenyl-tetrazolium bromide (MTT, ST316), N-Acetylcysteine (NAC, S0077), Z-VAD-FMK (C1202), and crystal violet staining solution (C0121) were obtained from Beyotime Biotechnology (Shanghai, China). 3-Methyladenine (3-MA, S2767) was purchased from Selleckchem (Houston, TX, USA). Anti-ATF4 (11815S), anti-BIP (3177S), anti-CHOP (2895S), anti-eIF2α (5324S), anti-Phospho-eIF2α (Ser51) (3398S), anti-Bax (41162S), anti-BCL2 (15071S), anti-Bcl-xL (2764S), anti-Puma (98672S), anti-LC3B (3868S), anti-SQSTM1/p62 (88588S), anti-Cytochrome c (4272S), anti-Caspase-3 (9662S), anti-PARP (9532S), and anti-β-Tubulin (2128S) antibodies were purchased from Cell Signaling Technology (Danvers, MA, USA).

### 4.2. Cell Lines and Culture

Human colorectal cancer cell lines HCT116 and HCT8 were obtained from the American Type Culture Collection (ATCC, Manassas, VA, USA). HCT116 and HCT8 cells were cultured in McCoy’s 5a (FBS, 10100147, thermofisher Scientific, Waltham, MA, USA) and RPMI 1640 (Cytiva, SH30022.01, Xcellerex, MA, USA) medium supplemented with 10% fetal bovine serum (FBS, 10100147, thermofisher Scientific, Waltham, MA, USA), respectively. All cells were cultured in an incubator with a humidified atmosphere of 5% CO_2_ at 37 °C.

### 4.3. Cell Viability Assay

The inhibitory effect of compound 275# on HCT116, HCT8, and normal adult colonic epithelial cell line FHC was determined by MTT assay. Cells were harvested at a density of 90% and counted and seeded into a 96-well plate (1 × 10^3^ cells per well) containing 200 μL complete medium. After incubation for 24 h, cells were treated with compound 275# at various concentrations (0, 0.39, 0.78, 1.56, 3.125, 6.25, 12.5, 25, and 50 μM) and incubated for 1, 2, 3, 4, and 5 days, respectively. Subsequently, 20 μL MTT solution (5 mg/mL) was added to each well and incubated with the cells for 4 h. The medium was discarded, and 150 μL dimethylsulfoxide (DMSO) was added to each well to dissolve the formazan. The absorbance was measured at 570 nm using a microplate reader (Bio-Tek, Winooski, VT, USA). All experiments were performed in triplicate. The cell viability of the HCT116, HCT8, and FHC cell lines was calculated using GraphPad^®^ Prism 9.0 software (Dotmatics, Boston, MA, USA).

### 4.4. Colony Formation Assay

For the colony formation assay, HCT116 and HCT8 cells were seeded into a 6-well plate at a density of 1000 cells per well supplemented with 2 mL complete medium, and the cells were subsequently incubated for 24 h. After exposure to compound 275# at concentrations of 0, 5, 10, or 20 μM for 14 days, the colonies formed were washed with cold phosphate-buffered saline (PBS), fixed with 4% paraformaldehyde for 25 min and then stained with 0.5% crystal violet for 30 min at room temperature. The plates were scanned and visualized using a Perfection V800 Photo scanning apparatus (Seiko Epson Corporation, Tokyo, Japan). All statistical measurements were performed in triplicate.

### 4.5. Immunoblotting

Both HCT116 and HCT8 cells were treated with compound 275# alone at concentrations of 0, 5, 10, and 20 μM for 12 h or in combination with 5 mM NAC for 4 h. Subsequently, the harvested cells were lysed in radioimmunoprecipitation assay (RIPA) buffer (150 mM NaCl, 1% Nonidet P-40 (NP-40), 1% sodium deoxycholate, 0.1% sodium dodecyl sulfate (SDS), 25 mM Tris-HCl (pH 7.4), and 1 mM EDTA (pH 8.0)) supplemented with Halt™ Protease and Phosphatase Inhibitor Cocktail (Thermo Fisher Scientific, Waltham, MA, USA) on ice for 30 min. The lysates were centrifuged at 15,000× *g* for 20 min, and the supernatants were collected. Total protein concentrations of supernatants were quantified using BCA (Bicinchoninic acid) Kit (Beyotime, P0010, Shanghai, China). An equal amount of total proteins for each sample (30 μg) was loaded, separated by 10% and 15% SDS-polyacrylamide gel electrophoresis, and transferred to polyvinylidene difluoride (PVDF) membranes (MilliporeSigma, St. Louis, MO, USA). After that, the membranes were blocked with 5% fat-free milk in TBST for 2 h at room temperature, incubated with the indicated primary antibodies overnight at 4 °C, and then incubated with the IRDye^®^ 800CW goat anti-mouse IgG (H+L) or IRDye^®^ 680LT donkey anti-rabbit IgG (H+L) (LI-COR Biosciences) secondary antibody at room temperature in a dark environment for 1 h. Finally, antibody-bound membranes were washed 3 times in TBST each for 5 min, and immunoreactivity was visualized using an Odyssey Two-Color Infrared fluorescence Imaging System (LI-COR Biosciences, Lincoln, NH, USA).

### 4.6. Cell Apoptosis Analysis

The apoptosis of HCT116 and HCT8 was assayed by the Annexin V-FITC Apoptosis Detection Kit (C1062S, Beyotime, Shanghai, China) according to the manufacturer’s protocol. Briefly, cells were seeded into a 60 mm dish with a density of 2 × 10^6^ cells per dish and cultured overnight. After pretreatment with NAC (5 mM) or Z-VAD-FMK (50 μM) for 1 h, cells were exposed to different concentrations of compound 275# for 24 h. All treated samples were collected and washed with PBS and then incubated with annexin V-FITC and PI for 30 min at room temperature in the dark. Finally, cells were measured with flow cytometry, and the apoptosis ratio was analyzed using FlowJo 7.6.1 analysis software (Becton Dickinson, San Jose, CA, USA).

### 4.7. Mitochondrial Membrane Potential (MMP, ΔΨm) Assay

The ΔΨm of HCT116 and HCT8 was measured using an enhanced mitochondrial membrane potential assay kit with JC-1 (C2003S, Beyotime, Shanghai, China). Briefly, cells were seeded into a 60 mm dish with a density of 2 × 10^6^ cells per dish and cultured overnight. Cells were pretreated with 5 mM NAC for 1 h and then treated with compound 275# for additional 24 h until harvested for further incubation with the mitochondria-specific fluorescent dye JC-1 for 20 min at 37 °C. Then, JC-1 dye was removed and washed twice with JC-1 buffer. Finally, the ΔΨm of cells was measured by flow cytometry and analyzed by FlowJo 7.6.1 analysis software.

### 4.8. Determination of ROS Formation

The amount of cellular ROS was measured by Reactive Oxygen Species Assay Kit (S0033S, Beyotime, Shanghai, China). Briefly, cells were seeded into a 60 mm dish with a density of 2 × 10^6^ cells per dish and cultured for 24 h. Cells were pretreated with 5 mM NAC for 1 h or with DMSO alone (control) before adding compound 275# (up to 20 μM) for 2 h. After rinsing with PBS, the cells were incubated with 10 μM Dichloro-dihydro-fluorescein diacetate (DCFH-DA) for another 20 min in the dark at 37 °C, followed by flow cytometry detection to determine the level of cellular ROS. The analysis of cytometry data was achieved using FlowJo 7.6.1 software.

### 4.9. Fluorescence Observation of RFP-GFP-LC3B

For autophagy analysis, HCT116 cells stably expressed RFP-GFP-LC3B were constructed by lentivirus infection. Lentivirus packaging vectors (Pspax2, pMD2G) and RFP-GFP-LC3B were cotransfected into HEK293T cells using Lipo8000 Transfection Reagent (C0533, Beyotime, Shanghai, China) according to the manufacturer’s protocols. Viral particles were harvested after 48 h transfection and then added directly into HCT116 cells with the assistance of Polybrene (10 μg/mL). Subsequently, cells were selected by 5 μg/mL puromycin to obtain the cell-stable expression clones. The stable RFP-GFP-LC3B-expressing cells were pretreated with 3-MA (1 mM) or NAC (5 mM) for 1 h and further incubated with or without compound 275# for 12 h. The fluorescent LC3B dots were captured and analyzed by the high-content analysis system-operetta CLSTM (Perkinelmer, Waltham, MA, USA).

### 4.10. Statistical Analysis

All experiments were performed in triplicate. GraphPad Prism version 9.0 (GraphPad Software, San Diego, CA, USA) was used for statistical analysis. Data were presented as mean ±standard deviation, and the ANOVA method was used to compare differences between groups. *p* < 0.05 was considered to be significant.

## 5. Conclusions

In summary, a novel compound, termed compound 275#, was synthesized using pharmaceutical methods, and we found that it exhibits inhibitory effects on CRC cells. Interestingly, compound 275# promoted a mitochondria-mediated intrinsic apoptosis pathway and autophagy initiation due to the intracellular accumulation of ROS. Therefore, our findings favorably imply the antitumor mechanism of compound 275# and offer evidence of its potential as a promising new lead compound in the discovery of chemotherapeutic agents for CRC.

## Data Availability

The datasets used and/or analyzed during the current study are available from the corresponding author on reasonable request.

## Supplementary Materials

The following supporting information can be downloaded at: https://www.mdpi.com/article/10.3390/molecules28073211/s1, Figure S1: ^1^H NMR and ^13^C NMR spectra of compound 275#. Figure S2: Most of the ROS induced by compound 275# mainly originate from mitochondria. Figure S3: The protein level of p53 was not influenced by compound 275# and inferred that proapoptotic effect of compound 275# may be not mediated by p53. Figure S4: Pretreatment with Z-VAD-FMK can significantly improve cell viability and reduce the inhibitory effect of compound 275# on CRC cells.

## Sample Availability

Compounds synthesized in this present study are available from the authors.
