# Supplementary material for: Compound 275# Induces Mitochondria-Mediated Apoptosis and Autophagy Initiation in Colorectal Cancer Cells through an Accumulation of Intracellular ROS
Source: Molecules. 2023 Apr 4;28(7):3211. doi: 10.3390/molecules28073211 (PMC10095895; doi:10.3390/molecules28073211)
Supplement: Supplementary file 1 [file molecules-28-03211-s001.zip › molecules-2287355-supplementary.pdf]

Supplementary Materials

Compound 275# Induces Mitochondria-Mediated Apoptosis and Autophagy Initiation in Colorectal Cancer Cells through an Accumulation of Intracellular ROS

Contents:

Figure S1. ^{1}H NMR and ^{13}C NMR spectra of compound 275#.
Figure S2. Most of the ROS induced by compound 275# mainly originate from mitochondria.
Figure S3. The protein level of p53 was not influenced by compound 275# and inferred that pro apoptotic effect of compound 275# may be not mediated by p53.
Figure S4. Pretreatment with Z-VAD-FMK can significantly improve cell viability and reduce the inhibitory effect of compound 275# on CRC cells.

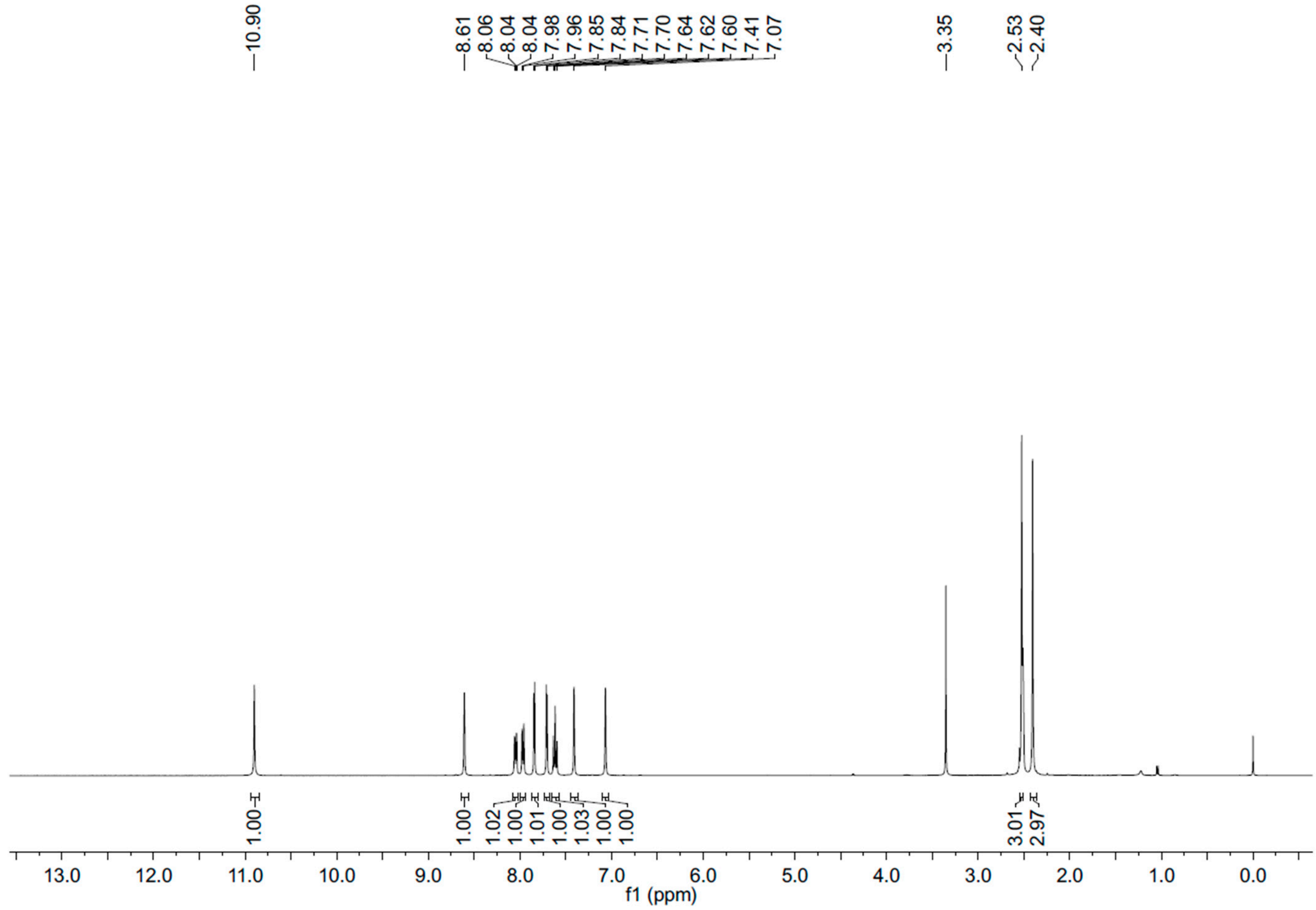

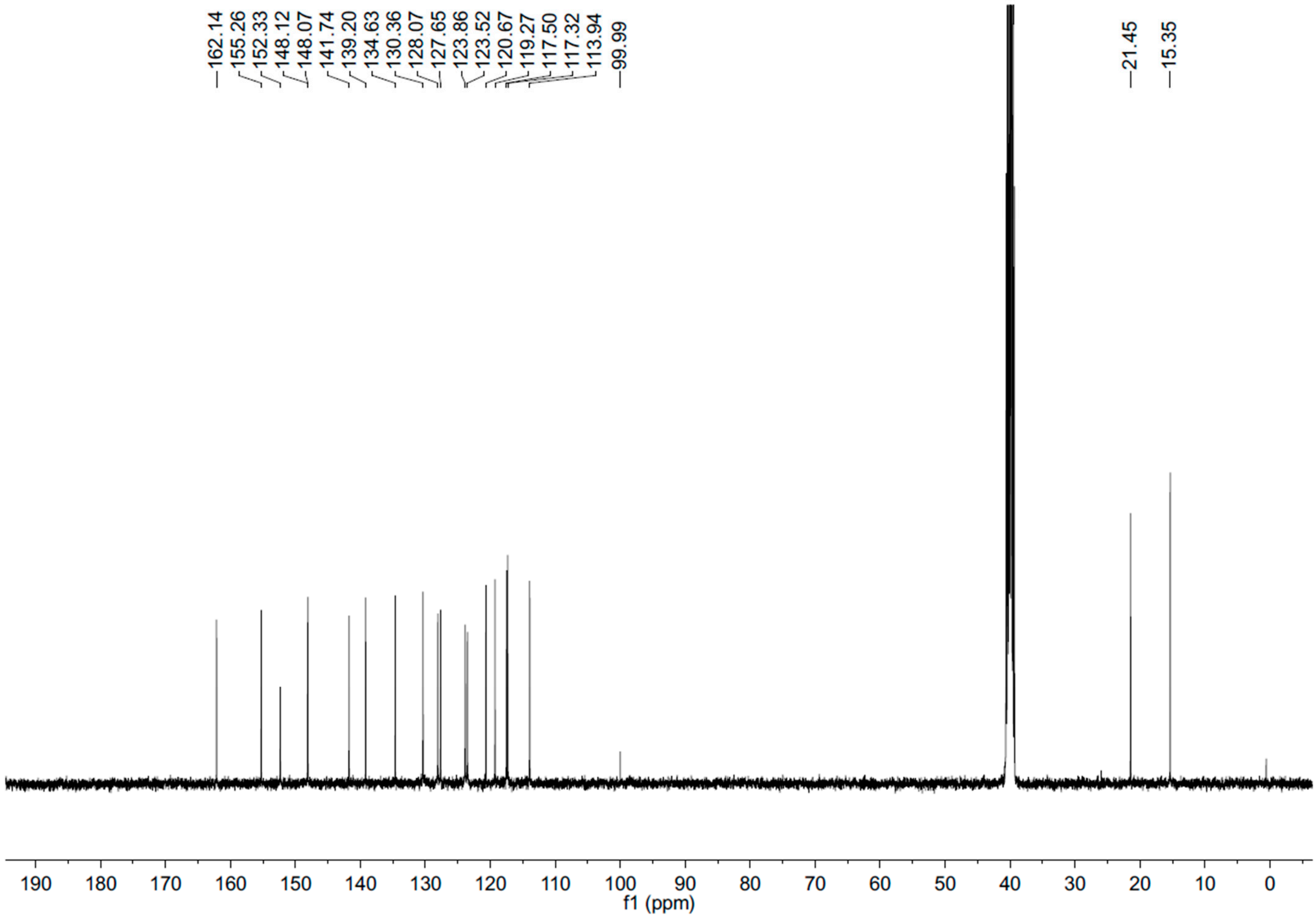

Figure S1. ^{1}H NMR and ^{13}C NMR of compound 275#.

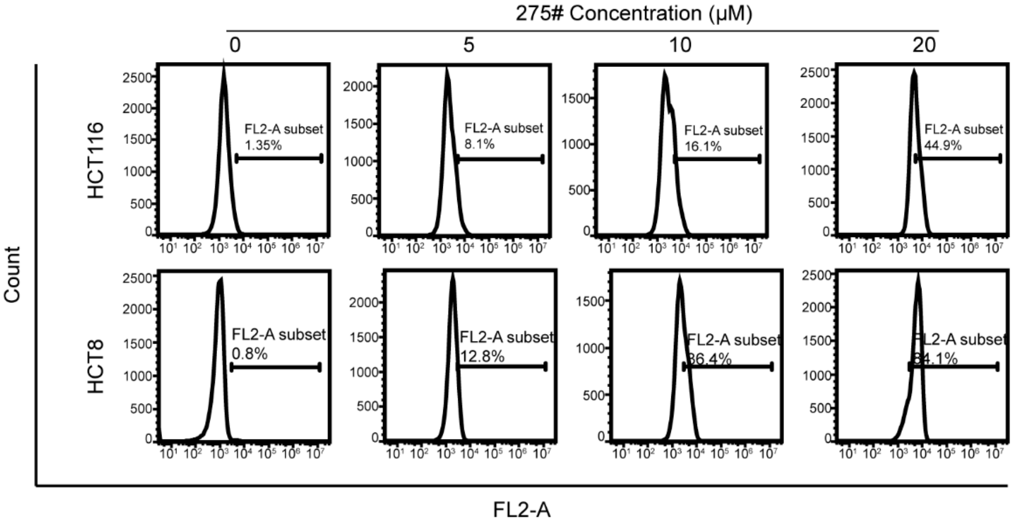

Figure S2. Most of the ROS induced by compound 275# mainly originate from mitochondria. HCT8 and HCT116 cells were treated with the indicated concentrations of compound 275# for 24 h in the presence of MITOSOX. Subsequently, flow cytometry analysis was used to detect the ROS.

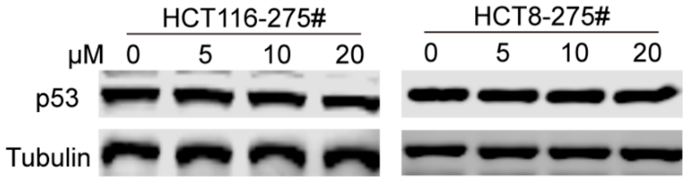

Figure S3. The protein level of p53 was not influenced by compound 275# and inferred that pro apoptotic effect of compound 275# may be not mediated by p53.

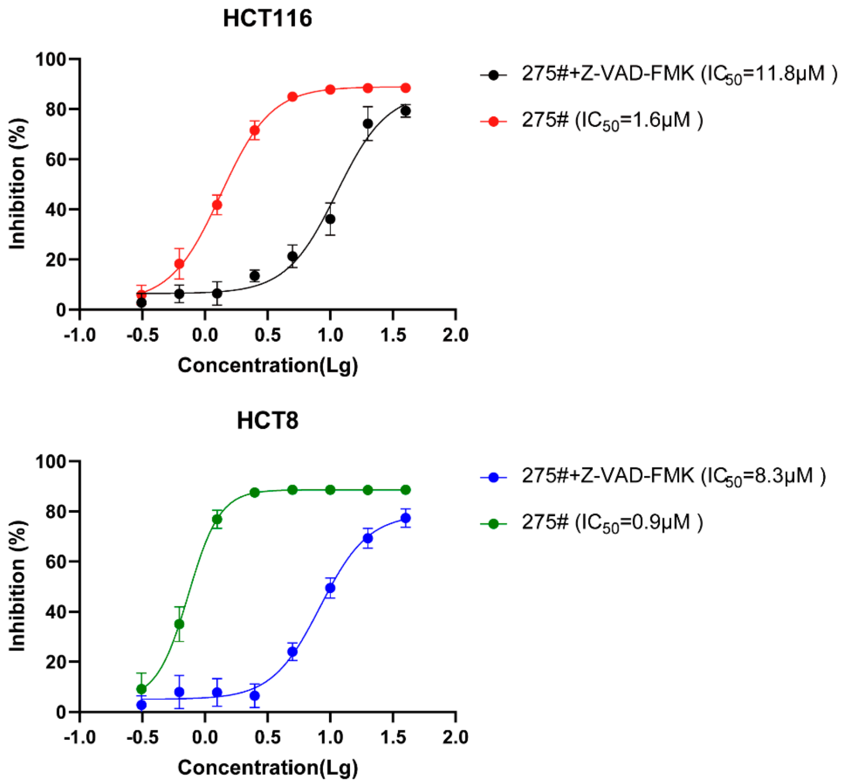

Figure S4. Pretreatment with Z-VAD-FMK can significantly improve cell viability and reduce the inhibitory effect of compound 275# on CRC cells.
